# Myelolipoma After Infliximab Treatment for Crohn's Disease

**DOI:** 10.14309/crj.0000000000000791

**Published:** 2022-07-01

**Authors:** Kevin Cesa, Elaine Leonard Puppa, Katayoun Eslami, Samra M. Blanchard, Runa D. Watkins

**Affiliations:** 1 Division of Pediatric Gastroenterology, Hepatology, and Nutrition, University of Maryland, Baltimore, MD; 2 Department of Pediatrics, University of Pittsburgh School of Medicine, Pittsburgh, PA

## Abstract

A 20-year-old woman with Crohn's disease receiving infliximab therapy presented to the emergency department with lower extremity swelling secondary to compression of the common iliac vein. On magnetic resonance imaging, an enlarging pelvic mass was identified. The pathology of the mass was consistent with myelolipoma. We believe this is the first case of myelolipoma in a patient on immunosuppression with infliximab.

## INTRODUCTION

Infliximab (IFX) is a common therapy for treating pediatric inflammatory bowel disease (IBD). In 2010, the Food and Drug Administration reported an increased risk of malignancy in children exposed to anti-TNF therapy,^1^ with subsequent studies provided conflicting results. Myelolipomas are rare benign neoplasms composed of adipose tissue and extramedullary hematopoiesis. To the best of our knowledge, this is the first reported case of myelolipoma in a patient on anti-TNF therapy.

## CASE REPORT

A 20-year-old White woman presented to the emergency department with a 3-week history of right lower extremity swelling. The patient had a history including Rett syndrome, Crohn's disease (CD), recurrent *Clostridium difficile* infections, and syndrome of inappropriate antidiuretic hormone secretion and adrenal insufficiency both secondary to prolong corticosteroid exposure. The patient was diagnosed with CD 9 years earlier when she presented with excessive gas, feeding intolerance, and hematochezia. Pathology showed chronic inflammation, architectural distortion, ulceration, and cryptitis consistent with IBD. The patient was induced with corticosteroids with mesalamine maintenance therapy. After 3 years of persistent symptoms, she underwent her most recent endoscopy that showed continued active disease. She was escalated to IFX (5 mg/kg every 6 weeks) and was able to achieve clinical, biochemical, and radiographical remission with no evidence of active disease on her most recent imaging 2 years earlier.

**Figure 1.**
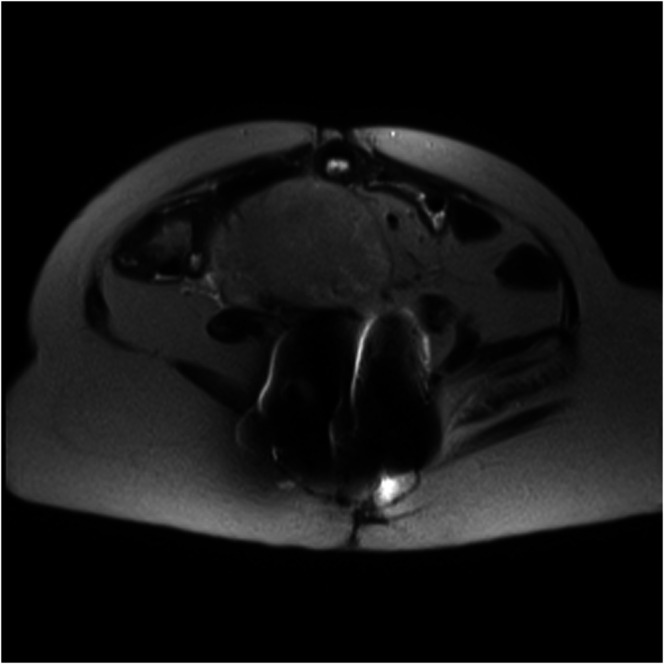
An enlarging pelvic mass on abdominal and pelvic magnetic resonance imaging.

An abdominopelvic CT showed a large heterogeneous solid right pelvic mass compressing the common iliac vein, which was not present on her most recent imaging. A transabdominal ultrasound identified a solid homogeneously echogenic right adnexal mass with minimal internal vascularity. The patient was transferred to the pediatric gastroenterology service, where a subsequent abdominal and pelvic magnetic resonance imaging with contrast displayed an enlarging pelvic mass (Figure 1). Pediatric oncology recommended surgery.

**Figure 2.**
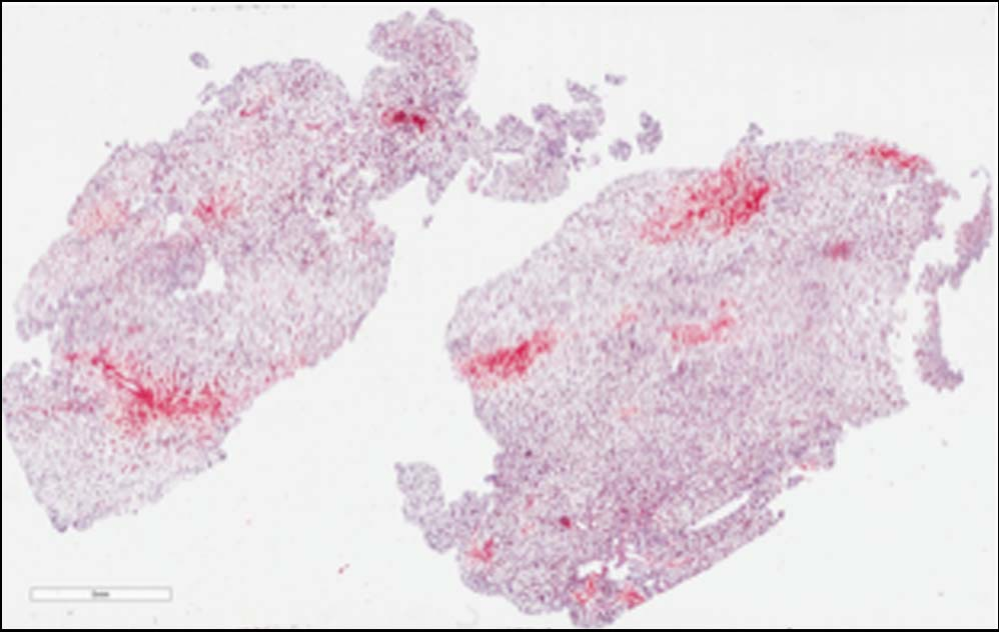
Retroperitoneum tumor biopsy consistent with myelolipoma: microscope low-power field.

**Figure 3.**
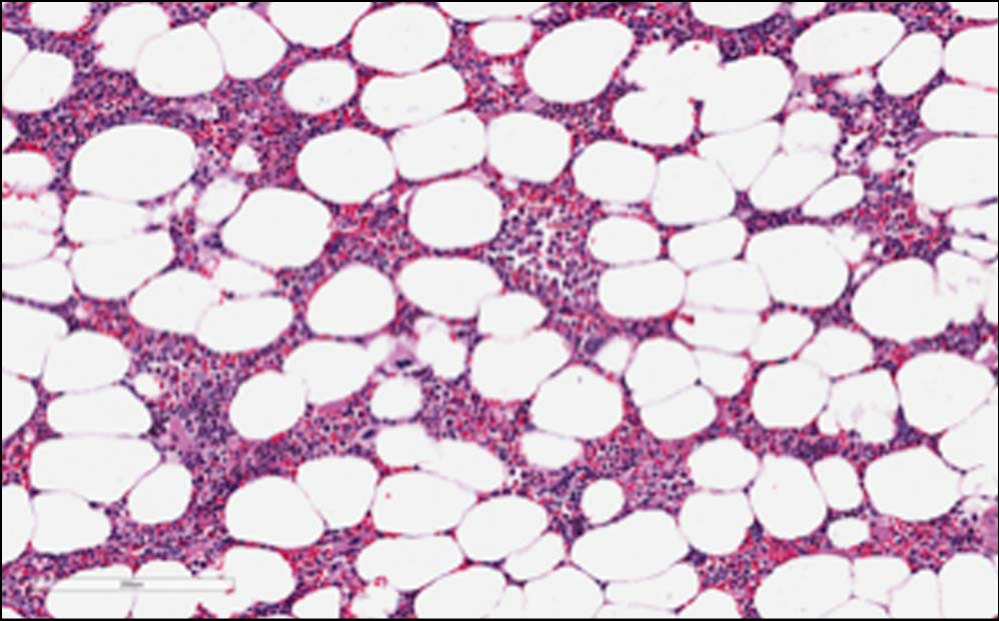
Retroperitoneum tumor biopsy consistent with myelolipoma: microscope high-power field.

Surgeons performed an exploratory laparotomy, identifying a necrotic-appearing mass in the retroperitoneum within the right aspect of the mesentery of the rectosigmoid colon. The surgery was terminated after near-total resection of the tumor (10 × 8 × 2 cm) because of the proximity to underlying pelvic vessels. The tumor was consistent with myelolipoma on pathology (Figures 2 and 3). After discussion with the family, IFX infusions were continued because the patient was able to achieve clinical remission on IFX monotherapy and the mass was not malignant. To date, the patient has remained in clinical and biochemical remission with no evidence of return of myelolipoma.

## DISCUSSION

IFX is a monoclonal antibody against anti-TNF α, first licensed for pediatric use in 2006. IFX has improved outcomes by inducing and maintaining remission in patients with IBD.^2^ The long-term safety of anti-TNF therapy continues to be of concern because the Food and Drug Administration has reported an increased risk of malignancies in children exposed to anti-TNF agents.^1^ The most common reported malignancies with anti-TNF therapy are lymphomas including non-Hodgkin, Hodgkin, and hepatosplenic T-cell.^3^ Benign tumors reported in patients with IBD include dermatofibroma, dermal nodules, and inflammatory pseudotumor of the liver.^4,5^ Similarly, benign tumors in patients on anti-TNF therapy are rare, with case reports of salivary tumors, papillary cystadenoma lymphomatosum, and pleomorphic adenoma.^6^ In addition, a minority of patients on anti-TNF therapy will paradoxically develop worsening inflammation with exacerbation of their chronic inflammatory disease or new-onset inflammatory/autoimmune disease including systemic lupus erythematosus, psoriasis, cutaneous vasculitis, and interstitial lung disease.^7,8^ A proposed mechanism for this inflammatory response is disruption of immune system homeostasis by anti-TNF therapy blockage of the TNF receptor type 1 resulting in the loss of macrophage inhabitation.^9^

IBD is associated with intestinal (colorectal carcinoma, small bowel adenocarcinoma, cholangiocarcinoma, and intestinal lymphoma) and extraintestinal (lymphoma, leukemia, cervical cancer, and melanoma) malignancies.^3,10^ There is concern that chronic immunosuppression from anti-TNF therapy may promote neoplasms, but a clear association has not been identified. Studies are confounded exposure to thiopurines, where there is an established risk of lymphoma and skin cancer. A 2017 meta-analysis from DEVELOP, the largest prospective cohort study of long-term safety outcomes in pediatric patients with IBD, did not show an association between IFX and malignancy.^10^ This conflicts with a 2011 Kaiser Permanente IBD Registry study that showed that anti-TNF therapy had a significantly increased rate of lymphoma with or without thiopurine use.^11^ A 2012 retrospective cohort and nested case-control study using administrative data from the LifeLink Health Plan Claims Database found that the rate of melanoma was increased by the use of biologics.^12^ In 2016, the European Crohn's and Colitis Organization concluded that there were insufficient data to suggest that anti-TNF agents alone increased the risk of lymphoproliferative disorders or solid tumors.^13^

Myelolipomas are benign neoplasms commonly found in the adrenal gland (90%-85%) composed of elements of adipose tissue and extramedullary hematopoiesis.^14,15^ The median age of presentation is 66.5 years, with only 2 pediatric cases in the literature.^16,17^ Congenital adrenal hyperplasia is associated with 10% of cases of adrenal myelolipoma while other endocrine disorders including hypercortisolism, primary aldosteronism, hypogonadism, primary hyperparathyroidism, and pheochromocytoma occur in 7.5% of other cases.^14^ Myelolipomas are associated with organ transplants, with immunosuppression cited as a risk factor.^18^ The most common cited mechanism for the development of myelolipoma is chronic stimulation of ectopic adrenal or hematopoietic stem cells from chronic inflammation, endocrine disorder, necrosis, and hypertension that results in metaplastic changes.^19^

Rett disease is a neurodevelopmental disorder caused by mutations in the X-linked gene methyl-CpG-binding protein 2, resulting in a range of phenotypes including regression of previously acquired speech and motor skills, repetitive hand movements, breathing irregularities, and seizures.^20^ We were unable to identify any previous case reports of myelolipomas in patients with Rett syndrome, IBD, CD, IFX, anti-TNF therapy, or syndrome of inappropriate antidiuretic hormone secretion.

## KEY POINTS

- Risks and possible associations with anti-TNF therapy (heart failure, sensitivity reactions, infection risk, malignancy, and paradoxical inflammation) should be discussed before starting the medication.^2^
- Owing to the unclear risk of anti-TNF therapy with malignancies, patients should be monitored for these diseases and unexpected outcomes should be reported.
- Myelolipomas are rare benign neoplasms that should be considered in patients with risk factors including endocrine disorders, immunosuppression, and chronic inflammation.

## DISCLOSURES

Author contributions: K. Cesa and K. Eslami wrote and edited the article and reviewed the literature. EL Puppa wrote and edited the article. SM Blanchard edited the article and approved the final article. RD Watkins edited the article, approved the final article, and is the article guarantor.

Financial disclosure: The authors have no disclosures to report.

Informed consent: Informed consent was obtained for this report.

Previous presentation: Presented at 2018 NASPGHAN Annual Meeting, October 10, 2018; Hollywood, Florida.
